# Alleviation of Inflammatory Conditions Caused by Extremely Low-Frequency Electromagnetic Field Exposure by *Panax ginseng*

**DOI:** 10.1155/mi/7870941

**Published:** 2025-11-21

**Authors:** Jee Yeon Choi, Ji Soo Lee, Seo An Lee, Hui Won Moon, So-Young Park, Kwang Woo Hwang

**Affiliations:** ^1^Department of Global Innovative Drugs, College of Pharmacy, Chung-Ang University, 84 Heukseok-ro, Dongjak-gu, Seoul 06974, Republic of Korea; ^2^College of Pharmacy, Dankook University, 119 Dandae-ro, Dongnam-gu, Cheonan-si, Chungnam 31116, Republic of Korea

**Keywords:** anti-inflammation, apoptosis, ELF-EMF, ginsenosides, macrophage

## Abstract

**Background:**

Electronic devices such as cellphones, microwaves, and other household devices are known to emit electromagnetic waves. As such, it creates an environment that may disrupt homeostasis. Ginsenosides in red ginseng is a Korean herb that is known for their anticancer, anti-inflammatory, and antidiabetic properties. This study aims to assess the therapeutic properties of ginsenosides in extremely low-frequency electromagnetic field exposure (ELF-EMF) environment.

**Materials and Methods:**

To observe the anti-inflammatory effects of ginsenosides, lipopolysaccharide (LPS) and ELF-EMF-induced RAW 264.7 cells were treated with 14 ginsenosides. Here, the production and gene expression of pro-inflammatory cytokines such as TNF-α, IL-6, IL-1β, and nitric oxide (NO) were determined. Furthermore, neuronal apoptosis was examined as this may be induced by excessive secretion of pro-inflammatory cytokines and increased calcium influx.

**Results:**

RAW264.7 cells exposed to ELF-EMF showed an increase in NO production and pro-inflammatory cytokines. Moreover, under ELF-EMF exposure, translocation of NF-kB increased and NFAT2, due to elevated calcium influx, increased as well. These inflammatory responses were alleviated by the ginsenosides and among the 14 ginsenosides, ginsenoside Rd had the most potent anti-inflammatory effect.

**Conclusion:**

Ginsenosides alleviate inflammation induced by ELF-EMF by downregulating inflammatory-related cytokines and proteins. It also had an effect on decreasing nerve cell apoptosis by reducing inflammatory response.

## 1. Introduction

Electromagnetic fields are a form of radiation that is grouped through coupling of electric and magnetic fields. These radiation fields may be produced by man-made devices such as mobile phones, microwaves, televisions, and more. Magnetic fields with frequencies up to 300 Hz are commonly called extremely low-frequency electromagnetic fields (ELF-EMFs) [1] and equipment found in a household usually produces 50–60 Hz, giving a possibility of being exposed to ELF-EMF [2]. Consistent exposure to such frequency may lead to functional changes in the human body, as stated by the World Health Organization, ELF-EMF is considered as a group 2B carcinogen, as there have been reports associating ELF-EMF exposure to childhood leukemia [3]. Several studies regarding exposure to ELF-EMF have been done in order to observe expression of inflammatory factors. Mice induced with ELF-EMF showed an increase in phagocytic activity of macrophages and production of superoxide radicals [4]. This may be due to stimulation of the NADH pathway, inducing superoxide anion radicals and ROS production [4, 5]. Our previous studies have shown the effects of ELF-EMF on T cells, wherein, exposure to such frequencies elevated the differentiation of T cells to T helper subsets 9 and 17. IL-9, generated by Th9, is known to enhance the activation of mast cells and secrete inflammatory cytokines [6]. For Th17, EMF exposure upregulated the expression of major Th17 markers such as IL-17, which is a pro-inflammatory cytokines known to cause several autoimmune diseases and RORγT, a key transcription factor responsible for the secretion of IL-17 cytokine [7]. With these studies, we were able to observe the inflammatory response caused by the exposure of ELF-EMF, which may be harmful. Contrary to these, several other studies showed exposure to electromagnetic fields showed no significant risk [3], which shows further studies should be conducted about the risks of ELF-EMF.

Korean red ginseng is a common herb known for its traditional function in improving immunity. The active ingredient responsible for the anticancer, anti-inflammatory, antiobesity, and antidiabetic effects of the red ginseng is the saponin compound known as ginsenoside [8–10]. Currently, the effects of ginsenosides on the immune responses are being studied as these effects are known to modulate cytokine secretion in the innate immunity. For instance, ginsenoside Rb1 and compound K's effects in colitic mice were observed. It was shown that Rb1 and compound K suppressed the inflammation in the bowel of colitic mice by regulating the secretion of pro-inflammatory-mediated signaling through inhibition of IL-1 receptor-associated kinase 1 activation [4]. In the CNS, prolonged elevations of pro-inflammatory cytokines may stimulate neuronal damage and cognitive dysfunction. Neuronal apoptosis is a significant event in neuronal depletion, correlated to neurological disorders, which are modulated by pro- and antiapoptotic factors [11]. Effects of ginsenoside Rg3 in the hippocampus, responsible for the memory and learning in rats, decreased the cellular levels of TNF-α, IL-1β, and COX-2 [5, 6, 12]. The effects of ginseng on calretinin expression in mouse hippocampus followed by exposure to radiofrequency showed that ginseng has a neuroprotective effect by maintaining the Ca^2+^ homeostasis in the hippocampus through Ca^2+^ binding proteins [13–15]. Although efficacy of ginsenoside in maintaining calcium homeostasis has been observed, its effect on neuronal cell death influenced by cellular stress have yet to be discovered, therefore, the goal of this research is to observe the effects of ginsenoside on inflammation and neuronal apoptosis caused by ELF-EMF exposure using mouse macrophage, RAW 264.7, mouse neuronal cell line, PC12, and 14 ginsenosides.

## 2. Materials and Methods

### 2.1. ELF-EMF System

**Figure 1:**
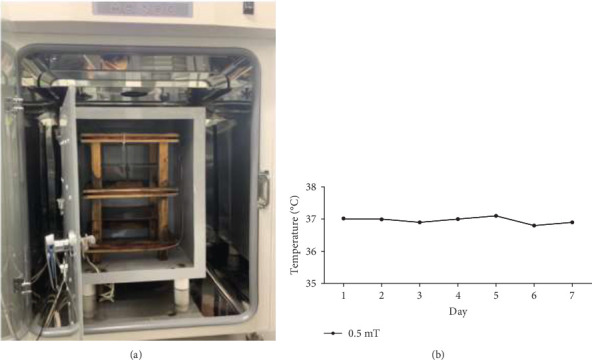
60 Hz 0.3 mT electromagnetic field exposure system. (A) ELF-EMF exposure system, which consists of an electromagnetic coil and a ferrite shield in a CO_2_ incubator. (B) Maintenance of temperature in the incubator.

The equipment for ELF-EMFs generation used in this study was designed and constructed by the Electrotechnology Research Institute (Dankook University, Cheonan, Republic of Korea). The 60-Hz ELF-EMFs were produced by a pair of Helmholtz coils with curves embedded in an open wooden square frame. Each coil was wrapped with a 1.0 mm diameter of insulated soft copper wire 200 times and was connected to a 220-V AC alternating power supply via a transformer. The central magnetic field strength was continuously maintained at 0.5 mT, which was monitored by a handheld Teslameter (Kanetec, Tokyo, Japan). The internal space of the incubator was shielded with an 80% nickel mu-metal chamber to keep uniformity by preventing external EMF (Figure 1A). The temperature in the incubator at 0.5 mT was maintained at 37 ± 0.5°C during the experimental period (Figure 1B).

### 2.2. Materials

Nine ginsenoside standards [Rb1, Rb3, Rd, Re, Rg1, Rg2(20S), Rg3(20S), Rh1(20S), Rh1(S)] were purchased from Ace EMzyme (Anseong, South Korea) and six ginsenoside standards [Rb2, Rc, Rf, Rg2(20R), Rg3(20R), Rh2(20R)] were from Ambo Institute (Daejeon, South Korea). Ginsenosides were dissolved in dimethyl sulfoxide from Sigma–Aldrich (St. Louis, MO).

### 2.3. Cell Culture

RAW 264.7 macrophage cells were procured from the American Type Culture Collection (Manassas, VA). The cells were cultured in Dulbecco's modified Eagle's medium (DMEM) (Cellgro, Manassas, VA) with fecal bovine serum (10%, heat-incubated, 35-015-CV, Cellgro), penicillin and streptomycin (100 U/mL, 0.1 mg/mL, 30-002-CI, Cellgro) and L-glutamine (2 mM, 25-005-CI Cellgro) at 37°C in a 5% CO_2_-humidified incubator.

### 2.4. Cell Viability Assay

To confirm the cytotoxicity of ginsenosides, 5 × 10^5^ cells of RAW264.7 were seeded in a 96-well plate and attached for 4 h. Followed by the treatment of 14 ginsenosides at specified concentrations and cultured overnight. After 24 h, the cultured cells were treated with 10 μL of 5 mg/mL MTT (3-(4, 5-dimethylthiazolyl-2)-2, 5-diphenyltetrazolium bromide) solution from Sigma–Aldrich (St. Louis, MO) and incubated the plate for 2 h. The supernatant was then removed, followed by adding 150 uL of DMSO to dissolve formazan, and the absorbance was measured at 570 nm using a microplate reader (Emax, Molecular Devices).

### 2.5. Nitric Oxide (NO) Assay

Chronically exposed (7 days) to the ELF-EMF or normal RAW264.7 cells were plated and attached overnight. After the incubation, supernatants were removed, and cells were pre-treated with 14 ginsenosides at 50 or 100 μM for 2 h. Then, 1 μg/mL of lipopolysaccharides (LPSs) from Sigma–Aldrich (St. Louis, MO) was treated and cultured for 24 h. The normal plate was placed in a CO_2_ incubator, and the EMF plate was placed in an ELF-EMF incubator and cultured under the same temperature, humidity, and CO_2_ concentration.

The supernatant was harvested and determined the NO production using Griess reagent from Sigma–Aldrich (St. Louis, MO). The same volume of the supernatant and dilution series of standard nitrite were mixed with Griess reagent and incubated for 15 min at 37°C in the dark. The absorbance was determined at 540 nm using a microplate reader.

### 2.6. Enzyme-Linked Immunosorbent Assay (ELISA)

Chronically exposed to the ELF-EMF or normal RAW 264.7 cells were cultured, supernatant was harvested, and concentrations of TNF-α, IL-6, and IL-1β were detected using ELISA. The 96-well microplates were coated with a capture antibody overnight at 4°C. The plate was incubated for an hour at room temperature with 3% BSA in PBS blocking solution. Samples and diluted solutions were introduced to the plate and incubated overnight at 4°C. After, the biotinylated antibody was added and incubated for 30 min at room temperature. Following the addition of Avidin-conjugated alkaline phosphatase from Jackson ImmunoResearch (Westgrove, PA). Lastly, the substrate solution was added (1 mg/mL 4-nitrophenyl phosphate disodium salt hexahydrate in 10% diethanolamine buffer). The plates were maintained for 5–30 min at room temperature before the addition of the stop buffer (1M NaOH). The absorbance was read at 405 nm using a microplate reader (Emax, Molecular Devices).

### 2.7. RNA Isolation and Quantitative Real-Time RT-PCR

RAW 264.7 cells, exposed to ELF-EMF or normal conditions, were stimulated with LPS in the presence of three ginsenosides, as described above. Cells were incubated for the optimal time for each gene. Total RNA was isolated using TRIzol from Invitrogen (Carlsbad, CA) and transcribed to cDNA for 1 h at 42°C in the cocktail, which contains MMLV-RT (Maloney murine leukemia virus reverse transcriptase, Promega, Madison, WI), 5X RT buffer, dNTPs (10 mM, 200 U), and oligo-dT primer (100 pmol). To determine mRNA levels of TNF-α, IL-6, IL-1β, and iNOS, quantitative PCR was performed using 2X iQ SYBR Green Supermix from Bio-Rad (Hercules, CA). cDNA was amplified under the following conditions: 95°C for 3 min followed by 45 cycles of 95°C for 10 s and 60°C for 30 s using a CFX Connect Real-time PCR Detection System from Bio-Rad (Hercules, CA). The PCR products were subjected to a melting curve analysis, and the comparative threshold method was used to estimate the relative level of mRNA in the experimental samples compared with the control. Gene expression was normalized to the expression levels of GAPDH.

### 2.8. Western Blot Analysis

Proteins from whole cells were extracted in RIPA buffer from Thermo Scientific (Rockford, IL) in the presence of protease inhibitor cocktail and phosphatase inhibitor cocktail. Nuclear and cytoplasmic proteins were extracted separately using the NE-PER Nuclear and Cytoplasmic Extraction Reagents kit from Thermo Scientific (Rockford, IL) according to the protocol supplied by the manufacturer. 30 μg of protein per lane was separated by 10% SDS-PAGE (sodium dodecyl sulfate-polyacrylamide gel electrophoresis) and transferred to polyvinylidene fluoride (PVDF) membranes from Bio-Rad (Hercules, CA). Blots were blocked with 5% skim milk and probed overnight with anti-IкBa, anti-NF-kB p65, anti-NFAT2, anti-p84, and anti-β-actin antibodies with 1:1000 dilution. The membranes were then incubated with a 1:5000 dilution of anti-mouse IgG or a 1:2000 dilution of anti-rabbit IgG HRP-linked secondary antibodies from cell signaling (Danvers, MA) for 2 h at RT. And the blots were visualized and captured using an immunoblot chemiluminescence detection system to a CCD camera system (Fusion Solo X, Vilber Lourmat).

### 2.9. Calcium Influx Assay

To detect intracellular Ca^2+^ mobilization of RAW264.7, which were exposed to ELF-EMF with or without ginsenosides, cells were processed with 3 μM of Fluo-4 AM (acetoxymethyl ester) from Invitrogen (Carlsbad, CA). In brief, harvested cells were washed twice using Ca^2+^ and Mg^2+^ free HBSS (Hanks Balanced Salt Solution) from Welgene (Gyeongsan, Republic of Korea) containing 20 mM HEPES from Cellgro (New York, USA). Cells were then incubated with HBSS buffer containing HBSS, 2.5 mM of fluo-4 AM in the presence of probenecid from Invitrogen (Carlsbad, CA) for 30 min at 37°C in 5% CO_2_. After staining, cells were washed twice again, followed by incubation for 20 min at RT. Cells were stimulated by ionomycin from Invitrogen (Carlsbad, CA) during measurements of Fluo-4 fluorescence using FACSCalibur and analyzed by CellQuest software. They are normalized based on their basal level.

### 2.10. Co-Culture

PC12 cells, rat pheochromocytoma cells, were procured from the Korean Cell Line Bank (KCLB, Seoul, Republic of Korea) and cultivated in RPMI 1640 medium supplemented with fetal bovine serum (10%, heat-inactivated), penicillin (100 U/mL), streptomycin (0.1 mg/mL), ʟ-glutamine (2 mM), and 2-mercaptoethanol at 37°C in 5% CO_2_. 2 × 10^5^ cells were plated in a 24-well plate for 8 h. In an insert-culture system, chronically exposed or normal RAW 264.7 cells which were attached for 4 h followed culturing with ginsenosides (200 μM) and LPS (1 μg/mL) for 4 h were seeded on to the same wells using cell culture inserts (membrane pore size 0.4 μm, BD Biosciences, Franklin Lakes, NJ). The plate was incubated for 1 day in the environment of ELF-EMF or normal.

### 2.11. Statistical Analysis

All experiments were done in triplicate, and results were expressed as mean ± standard deviation. The significance of data was determined by Student's *t*-test (*⁣*^*∗*^*p*  < 0.05; *⁣*^*∗∗*^*p*  < 0.01; and *⁣*^*∗∗∗*^*p*  < 0.001).

## 3. Results

### 3.1. RAW264.7 Macrophages Exposed to ELF-EMF Increases Inflammatory Factors

LPS activates macrophages via the Toll-like receptor 4 (TLR4), which then releases cytokines such as TNF-ɑ, IL-6, and IL-1β, and an enzyme known as NOS2, responsible for producing NO [16].

**Figure 2:**
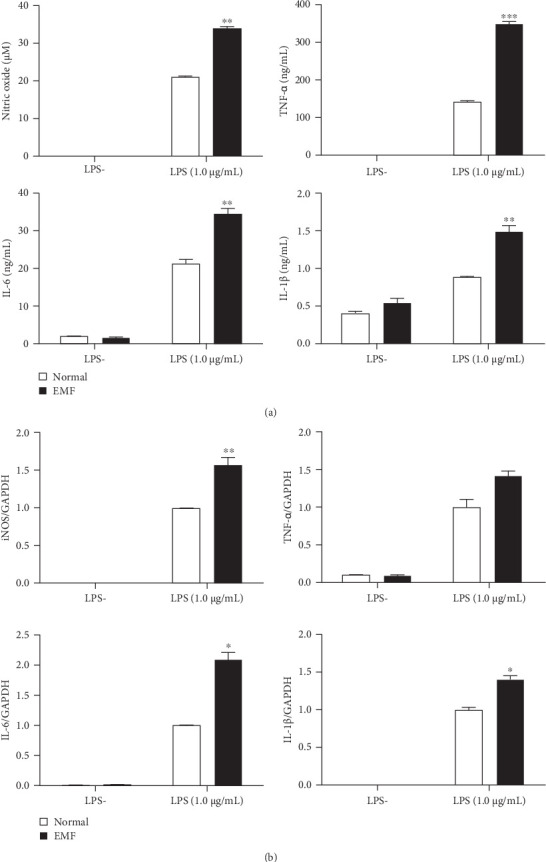
Biological changes in ELF-EMF exposed RAW 264.7 cells. (A) After 7 days of exposure to ELF-EMF, RAW 264.7 cells were cultured with or without 1 μg/mL of LPS. Nitric oxide production was observed using NO assay and TNF-ɑ, IL-6, and IL-1β production were detected using ELISA. (B) Gene expression of iNOS, TNF-ɑ, IL-6, and IL-1β was detected using RT-qPCR. The experiments were repeated three times. Values represent mean ± SD. *⁣*^*∗*^*p*  < 0.05, *⁣*^*∗∗*^*p*  < 0.01, and *⁣*^*∗∗∗*^*p*  < 0.001 comparing with the LPS untreated group (Student's *t*-test).

To assess the biological changes that occur under ELF-EMF environment, RAW 264.7 macrophages were exposed to ELF-EMF with or without LPS stimulation. Supernatants were obtained to observe NO production and pro-inflammatory cytokines such as TNF-ɑ, IL-6, and IL-1β. RAW 264.7 cells exposed to ELF-EMF with LPS stimulation showed a significant increase in pro-inflammatory cytokines and NO production while cells that were exposed to ELF-EMF without LPS stimulation showed no changes, suggesting stimulus is needed for an inflammatory response to occur (Figure 2A).

After confirming NO and inflammatory-mediated cytokine production via protein level, we observed the gene expression of iNOS, TNF-ɑ, IL-6, and IL-1β via real time PCR. RAW 264.7 cells exposed to ELF-EMF stimulated with LPS significantly increased the pro-inflammatory cytokines and iNOS expression compared to the non-stimulated group, indicating that LPS in combination with ELF-EMF stimulates an immune response from macrophages (Figure 2B).

### 3.2. Ginsenosides Inhibit NO Production in the ELF-EMF Environment

**Figure 3:**
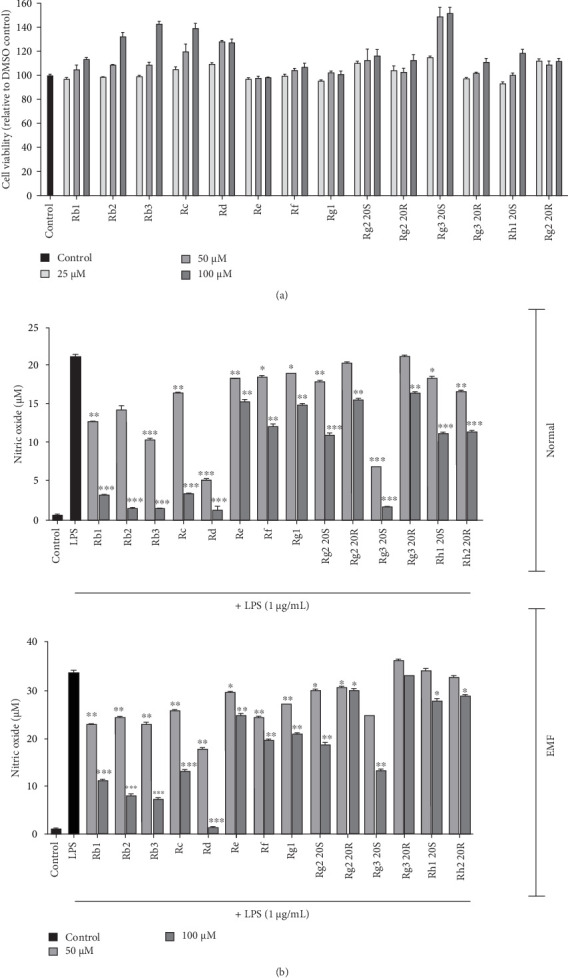
Ginsenosides suppress NO production in ELF-EMF exposed RAW 264.7 cells. (A) Cells were treated with different concentrations of ginsenosides for 24 h. Viability was quantitated by MTT assay. (B) RAW 264.7 cells were pre-treated with 14 ginsenosides at 50 or 100 μM. After 2 h, 1 μg/mL of lipopolysaccharides (LPS) was treated and cultured for 24 h. NO production was detected using NO assay. The experiments were repeated three times. Values represent mean ± SD. *⁣*^*∗*^*p*  < 0.05, *⁣*^*∗∗*^*p*  < 0.01, and *⁣*^*∗∗∗*^*p*  < 0.001 comparing it with the control group (Student's *t*-test).

To determine the cytotoxicity of the ginsenosides, a cell viability assay was conducted by treating RAW 264.7 cells with 14 ginsenosides in increasing concentrations. All ginsenoside samples showed 80% cell viability, thus, we proceeded to conduct further experiments using the following concentrations: 100, 50, and 25 μM (Figure 3A).

Ginsenosides are known to have an anti-inflammatory effect, inhibiting cytokine expression and NO production [17]. To confirm this anti-inflammatory effect, NO assay was performed using the same condition mentioned above. Results show that RAW 264.7 cells treated with ginsenosides at a concentration of 50 and 100 μM during culture, decreased NO production but significant reduction was observed in both the normal and EMF experimental groups at a concentration of 100 μM in Rb1, Rb2, Rb3, Rc, Rd, and Rg3 20S. Among these, Rd, in the concentration of 100 μM, appears to have a more potent anti-inflammatory effect (Figure 3B). Further experiments were conducted with the above-mentioned ginsenosides.

### 3.3. Ginsenoside Rd Significantly Inhibits the Production of Inflammatory Cytokines in the ELF-EMF Environment

Here we observed how ginsenosides were able to act as an anti-inflammatory mediator in response to LPS stimulation under ELF-EMF or normal environment. Pro-inflammatory cytokines such as TNF-α, IL-6, and IL-1β showed a significant decrease in secretion when macrophages were treated with ginsenosides Rb, Rd, and Rg3 20S.

**Figure 4:**
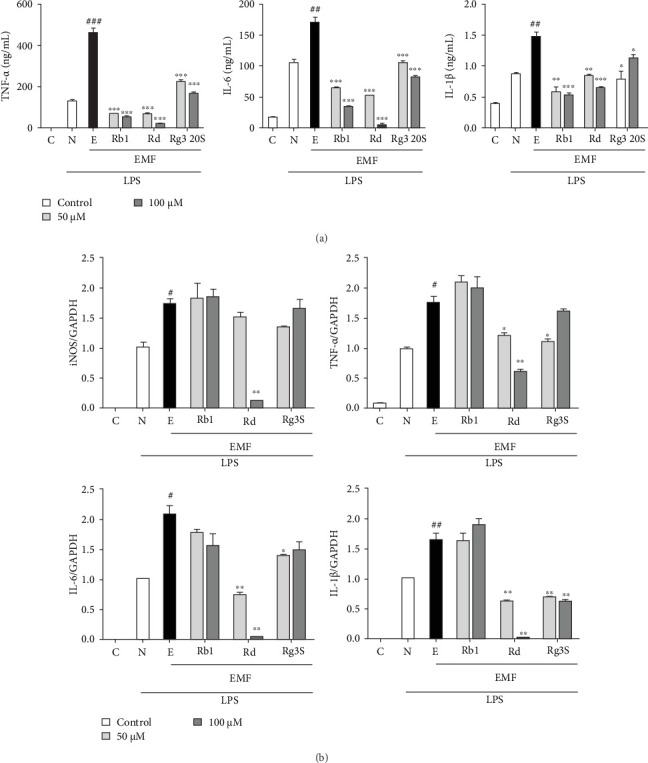
Inhibition of inflammatory cytokines and iNOS by ginsenosides. RAW 264.7 cells were treated with ginsenosides Rb1, Rd, or Rg3 20S at 50 or 100 μM. After 2 h, it was stimulated with or without LPS (1 μg/mL). (A) Secretion of inflammatory cytokines TNF-α, IL-6, and IL-1β was detected using ELISA. (B) Inflammatory cytokines and iNOS gene expression level were detected using RT-qPCR. The experiments were repeated three times. C represents the control group, N represents RAW264.7 cells pre-treated with LPS, and E represents RAW264.7 cells pre-treated with LPS and stimulated with ELF-EMF. Values represent mean ± SD. *⁣*^*∗*^*p*  < 0.05 and *⁣*^*∗∗*^*p*  < 0.01 comparing it with the ELF-EMF group (Student's *t*-test).

In the interest of confirming the inhibitory effects of ginsenosides' inflammatory response, macrophages, in ELF-EMF or normal environment, were stimulated with or without LPS and treated with 50 or 100 μM of ginsenosides during culture to observe the secretions of TNF-α, IL-6, and IL-1β. Pro-inflammatory cytokines were significantly increased when macrophages were exposed to the ELF-EMF but due to the inhibitory effects of ginsenosides Rb, Rd, and Rg3 20S, pro-inflammatory cytokines were decreased. Especially the anti-inflammatory effects of ginsenoside Rd showed a prominent decrease in the pro-inflammatory cytokine secretion (Figure 4A).

To assess the anti-inflammatory effects of the three selected ginsenosides at the gene level, the gene expression of the TNF-α, IL-6, IL-1β, and iNOS was observed using quantitative real time PCR. GAPDH was used as a standard housekeeping gene for quantification of the gene expression. TNF-α, IL-6, IL-1β, and iNOS gene expressions were increased when macrophages were exposed to the ELF-EMF but were significantly decreased in the ginsenoside Rd, Rg3 20S treated group (Figure 4B).

### 3.4. Ginsenoside Rd Inhibits Inflammatory Mediated Signaling Cascades

**Figure 5:**
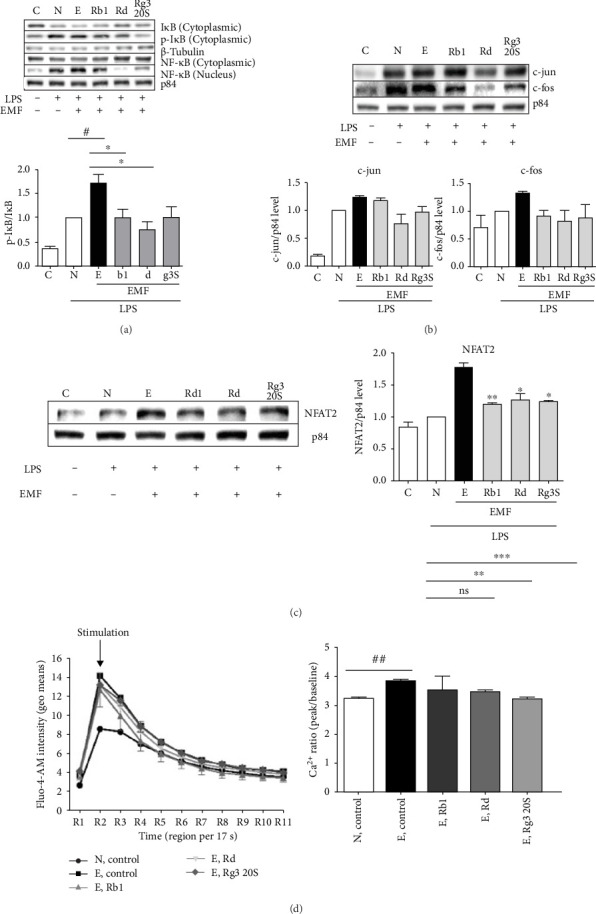
Ginsenosides inhibits inflammatory mediated signaling cascade. The expression of (A) NF-κB, (B) c-jun and c-fos, and (C) NFAT2, was detected by western blot analysis. The experiments were repeated three times. (D) RAW 264.7 cells were stimulated by 1 μg/mL LPS and cultured overnight with ELF-EMF. Calcium-indicator dye fluo-4 AM was added to evaluate calcium influx that follows when these cells are stimulated with ionomycin. Results were detected using flow cytometry. C represents the control group, N represents RAW264.7 cells pre-treated with LPS, and E represents RAW264.7 cells pre-treated with LPS and stimulated with ELF-EMF. Values represent mean ± SD. *⁣*^*∗*^*p*  < 0.05 and *⁣*^*∗∗*^*p*  < 0.01 and *⁣*^*∗∗∗*^*p*  < 0.001 compared with the ELF-EMF control group (Student's *t*-test).

NF-κB exists as a dimer composed of p65 and p50 proteins and is translocated from the cytoplasm into the nucleus when stimulated by a certain signal, activating transcription and inflammatory response signaling [18]. To further investigate this signaling pathway, cells were exposed to electromagnetic field and were treated with ginsenosides Rb1, Rd, or Rg3 20S at a concentration of 100 μM, and cultured cells were taken to confirm the expression of the NF-κB p65 protein in cytoplasmic protein and nucleus protein extraction. As shown in Figure 4A, NF-κB p65 protein expression in the nucleus was down-regulated in the ginsenosides-treated group compared to the ELF-EMF group. Ginsenoside Rd significantly decreased NF-κB p65 protein expression in the nucleus. With this, NF-κB signal transmission, which was activated by the electromagnetic field, was significantly decreased when treated with the three ginsenosides, reducing the translocation to the nucleus (Figure 5A).

The expression patterns of transcriptional complex AP-1, composed of c-Fos and c-Jun proteins, which are subfactors of MAPK signaling involved in inflammatory response signaling, were observed the same way as mentioned above. Results show that the expression of c-jun and c-fos in nucleus proteins increased upon ELF-EMF exposure and decreased in the ginsenoside Rd, Rg3 20S treated group. Activation of the inflammatory response mechanism in ELF_EMF environment increased the dimeric transcription factor AP-1. Signal transmission of AP-1 was suppressed by treatment of the three ginsenosides, resulting in anti-inflammatory and immunomodulatory effects (Figure 5B).

The NFAT2 protein is transcribed by forming a complex with the AP-1 protein to mediate various immune responses and is also known to be involved in producing infectious cytokines such as TNF-α. Calcium is known to be the second messenger of cells, and NFAT is controlled by calcium-calmodulin-dependent calcineurin signaling [19]. Expression pattern of NFAT2 under ELF-EMF exposure and regulatory effects of ginsenoside treatment were observed through western blot. As a result, the expression of NFAT2 protein increased under ELF-EMF environment and decreased in the ginsenoside treated group. A significant decrease was observed in the ginsenoside Rd treated group (Figure 5C).

Studies show that ELF-EMF environment increases voltage-gated Ca^2+^ [20]. To further validate the effects of ginsenoside in ELF-EMF environment, calcium influx assay was performed by using Fluo-4 AM dye, which can be used to detect intracellular free calcium. Under the ELF-EMF environment, influx of calcium increased and in contrary to this, calcium levels were decreased when treated with ginsenosides Rb1, Rd, and Rg3 20S (Figure 5D). This observation gives us the idea that ginsenosides have the effect of blocking calcium influx.

### 3.5. Ginsenosides Inhibit Inflammatory Macrophage Induced Nerve Cell Death

**Figure 6:**
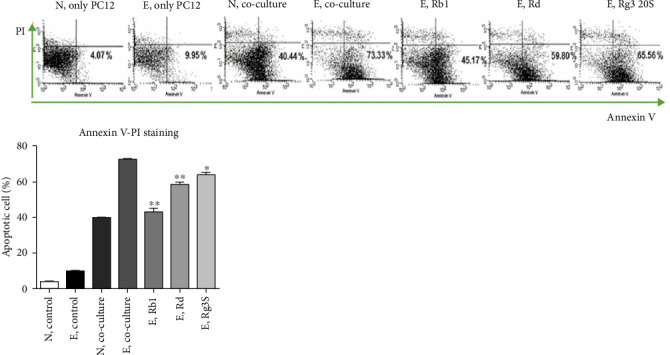
Ginsenosides increase cell survival of PC12. Co-culture of RAW 264.7 cells and PC21 cells with or without LPS stimulation and ELF-EMF exposure. Apoptosis was observed by using Annexin V-PI staining and detected through FACS. The experiment was repeated twice. N represents normal control group. E represents ELF-EMF treated group.

Finally, to explore the effect of ginsenosides in ELF-EMF environment on nerve cells, co-culture of PC12 cells and LPS stimulated RAW264.7 cells were performed and apoptosis was observed using Annexin V-PI staining through flow cytometry. Annexin V-FITC is a method, which monitors the progress of apoptosis by using the principle of binding with phosphatidylserine, which is usually translocated to the outer part of the plasma membrane and conjugated with fluorochromes [21]. First, PC12 cells were observed in normal conditions. Next, PC12 cells underwent ELF-EMF exposure to observe the increase in apoptotic cells. Thereafter, PC12 cells were co-cultured with LPS pre-treated RAW264.7 cells in normal conditions and ELF-EMF environment. Subsequent to this, the ELF-EMF stimulated PC12 and RAW264.7 co-culture were treated with ginsenosides Rb1, Rd, and Rg3 20S. FACS results indicated that co-culture of macrophages and neurons with active inflammatory reactions by stimulating with LPS induces apoptosis of nerve cells and apoptotic cells were further increased when exposed to ELF-EMF. In addition, ginsenoside Rb1, Rd, and Rg3 20S treatments decreased apoptotic cells and a significant decrease was observed in the effect of ginsenoside Rg3 20S, signifying that ginsenosides have the effect of inhibiting nerve cell death and reducing inflammatory reactions (Figure 6).

## 4. Discussion

Electromagnetic fields are usually given off by electrical appliances that are normally found in a household [4]. Studies found that perpetual exposure to ELF-EMF increases inflammatory cytokines, causing several risks including neuroinflammatory diseases [3, 13, 14]. Previous studies have shown that RAW 264.7 cells are activated under ELF-EMF condition and release of pro-inflammatory cytokines is further increased when treated with LPS at different concentrations. There was also a dramatic increase in NO production when LPS stimulated RAW 264.7 cells were exposed to ELF-EMF. These findings suggests that ELF-EMF may enhance macrophage activation above the normal state. Current studies have yet to discover whether continuous exposure to ELF-EMF is harmful, therefore, our study aims to observe the impact of ELF-EMF on macrophage cell line RAW 264.7, surveilling expression of inflammatory cytokines such as TNF-α, IL-6, and IL-1β, NO production, and calcium channel activity.

Under ELF-EMF condition, macrophage cell line RAW 264.7 cells showed an increase in inflammatory cytokines and NO production compared to the control group. Similar with these results, ELF-EMF activated NFAT signaling pathway by promoting Ca^2+^ voltage-gated channels, increasing NFAT2 translocation to the nucleus, leading to an upregulation of pro-inflammatory genes such as TNF-α, IL-6, and iNOS [13, 19]. Under these conditions, it may be possible that overexpressed calcium ion channel caused by ELF-EMF disrupts the calcium homeostasis which causes an accumulation of ROS. This activates p53, a nuclear transcription factor that enhances the expression of pro-apoptotic genes specifically Bax, NOXA, Puma, and Bak and may also form inhibitory complexes to anti-apoptotic genes like Bcl-2 and Bcl-XL [22]. ROS is also known to induce phosphorylation of JNK which in turn, activates transcription factors like SMAD3 and can also translocate to the nucleus to activate c-Jun. This increases the possibility of cellular apoptosis. To confirm whether ELF-EMF do cause cellular apoptosis through this signaling pathway, we examined the rate of cell apoptosis via annexin V staining and were able to observe the increase in cell apoptosis under this condition.

Our previous study investigated the antioxidant effect of resveratrol and the effect of ELF-EMF on an antioxidant-related gene known as Prx-1. What came to our attention was that resveratrol had little to no effect in reducing the inflammation caused by ELF-EMF and Prx-1 gene knockdown also exhibited an increased expression of pro-inflammatory cytokines and NO production. We hypothesized that ELF-EMF may inhibit the antioxidant effect of Resveratrol or that the excessive inflammation caused by ELF-EMF disrupts the resveratrol from inducing antioxidative effects [4]. Ginsenoside has been studied for its anti-inflammatory and antioxidative effects on an inflammatory setting. Treatment with certain ginsenosides is known to alleviate inflammatory conditions, but ginsenosides' effects in an ELF-EMF environment have yet to be studied, so our aim was to observe anti-inflammatory and antioxidative properties of ginsenosides in the inflammatory setting caused by ELF-EMF and since it has been mentioned above that ELF-EMF inhibited the antioxidative function of resveratrol we also wanted to observe whether this applies to ginsenosides as well.

To observe the efficacy of ginsenosides in alleviating inflammatory response, RAW 264.7 macrophages were treated with 14 ginsenosides [4, 9, 10]. Ginsenosides exerted a notable anti-inflammatory effect on ELF-EMF-exposed RAW 264.7 cells by downregulating pro-inflammatory cytokines and reducing NO production. In particular, ginsenoside Rd significantly suppressed NO production. ELF-EMF exposure promoted phosphorylation and nuclear translocation of NF-κB, elevated the expression of c-jun and c-fos, and enhanced calcium channel activity; specifically, these inflammatory responses were modulated by treatment with ginsenosides Rb1, Rd, and Rg3 20S. Furthermore, neuronal cells co-cultured with RAW 264.7 cells were subjected to ELF-EMF exposure. In this context, neuronal cells known as PC12 cells showed an increased rate of apoptosis; however, these effects were mitigated upon treatment with ginsenosides with ginsenoside Rg3 20S exhibiting the most significant ameliorative effect. Although studies are still ongoing, ginsenosides, particularly Rd, and Rg3 20(s) showed a high efficacy in alleviating inflammation state. Rd is known to inhibit NF-κB more effectively than other ginsenosides due to its reduced glycosylation and high lipophilicity, which makes it easier to penetrate the lipid membrane [23–25]. But compared to Rd, Rg3 20(s) exhibited a stronger anti-inflammatory effect due to higher lipophilicity and stronger inhibition of the NF-κB pathway and activation of AMPK pathway, which restores cellular redox balance [24, 26]. Collectively, these findings demonstrate the efficacy of ginsenosides in mitigating inflammatory responses under ELF-EMF exposure.

**Figure 7:**
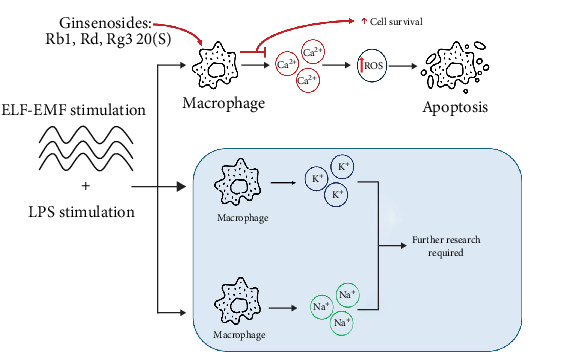
Effect of ELF-EMF exposure in macrophages. ELF-EMF mediates calcium ion channels in macrophages, increasing ROS activation, which leads to apoptosis. Further research is required for voltage-gated ion channels of sodium and potassium.

Further research is needed to observe the biological impact of ELF-EMF as the effects of ELF-EMF in different voltage-gated ion channels have yet to be studied. Observation of multiple ion channels such as Na^+^, K^+^, etc., may give off a different effect, which may also affect cellular metabolism (Figure 7).

## 5. Conclusion

This study demonstrates the negative impact of ELF-EMF on immune cells and the positive effect of ginsenosides. ELF-EMF increased pro-inflammatory cytokines such as TNF-α, IL-6, and IL-1β, and increased NO secretion, while ginsenosides significantly reduced the inflammation and rate of apoptosis. These findings show that the inflammatory setting caused by ELF-EMF may be negated by ginsenosides rather than resveratrol, as ginsenosides showed a higher probability with regards to anti-inflammatory and anti-oxidative effect. Targeting voltage-gated ion channels such as Na^+^ and K^+^ might give us a new way to observe the impact of ELF-EMF in immune cells.

## Data Availability

The data that support the findings of this study are available from the corresponding author upon reasonable request.

## Nomenclature

CNS: Central nervous system
ELF-EMF: Extremely low-frequency electromagnetic field exposure
ELISA: Enzyme-linked immunosorbent assay.
